# Effect of the Solvent Temperatures on Dynamics of Serine Protease Proteinase K

**DOI:** 10.3390/ijms17020254

**Published:** 2016-02-19

**Authors:** Peng Sang, Qiong Yang, Xing Du, Nan Yang, Li-Quan Yang, Xing-Lai Ji, Yun-Xin Fu, Zhao-Hui Meng, Shu-Qun Liu

**Affiliations:** 1 Laboratory for Conservation and Utilization of Bio-Resources, Yunnan University, Kunming 650091, China; speng431@163.com (P.S.); yq915702251@163.com (Q.Y.); duxingok@gmail.com (X.D.); yangnan138@163.com (N.Y.); ylqbioinfo@gmail.com (L.-Q.Y.); rich@ynu.edu.cn (X.-L.J.); yunxin.fu@uth.tmc.edu (Y.-X.F.); 2 Laboratory of Molecular Cardiology, Department of Cardiology, the First Affiliated Hospital of Kunming Medical University, Kunming 650032, China; 3 College of Agriculture and Biological Science, Dali University, Dali 671003, China; 4 Human Genetics Center, School of Public Health, the University of Texas Health Science Center, Houston, TX 77030, USA; 5 Key Laboratory for Tumor molecular biology of High Education in Yunnan Province, School of Life Sciences, Yunnan University, Kunming 650091, China

**Keywords:** solvent mobility, hierarchical dynamics of proteins, free energy landscape, conformational sampling

## Abstract

To obtain detailed information about the effect of the solvent temperatures on protein dynamics, multiple long molecular dynamics (MD) simulations of serine protease proteinase K with the solute and solvent coupled to different temperatures (either 300 or 180 K) have been performed. Comparative analyses demonstrate that the internal flexibility and mobility of proteinase K are strongly dependent on the solvent temperatures but weakly on the protein temperatures. The constructed free energy landscapes (FELs) at the high solvent temperatures exhibit a more rugged surface, broader spanning range, and higher minimum free energy level than do those at the low solvent temperatures. Comparison between the dynamic hydrogen bond (HB) numbers reveals that the high solvent temperatures intensify the competitive HB interactions between water molecules and protein surface atoms, and this in turn exacerbates the competitive HB interactions between protein internal atoms, thus enhancing the conformational flexibility and facilitating the collective motions of the protein. A refined FEL model was proposed to explain the role of the solvent mobility in facilitating the cascade amplification of microscopic motions of atoms and atomic groups into the global collective motions of the protein.

## 1. Introduction

Proteins are important bio-macromolecules because they perform a vast variety of functions and involve virtually every process within cells. However, proteins are not static entities but undergo complex internal motions, which are essential for their functions [1,2]. Therefore, a deeper understanding of protein functions needs to investigate their dynamic behaviors.

In a protein–solvent system or under physiological conditions, protein molecules are immerged in an aqueous solvent and thus the dynamic behaviors of the water molecules and the protein–solvent interactions are fundamental to the structural, dynamic, and functional properties of the proteins [3,4,5,6,7], including facilitating protein folding [8,9], maintaining structural stability/flexibility [10,11,12], mediating protein–ligand binding [13,14], accelerating enzymatic catalysis [15,16], and inducing protein glass transition [17,18,19].

The FEL, which is defined as a function describing the conformational states/substates of a protein with the corresponding Gibbs free energy of the protein–solvent system, is a seminal concept in characterizing and explaining many aspects of the protein dynamics and functions [2,20,21,22,23]. Due to an absolute advantage of the water molecules in terms of both quantity and mass compared to the solute molecules, the changes in entropy and enthalpy of the solvent will make a substantial contribution to the change in Gibbs free energy of the protein–solvent system [22,24,25], and the protein–solvent interactions also play a crucial role in shaping the FEL [26]. In the first fast stage of protein folding, *i.e.*, the hydrophobic collapse that results in the molten globule intermediate, the solvent entropy maximization contributes substantially to the lowering of the system free energy [27,28,29]. In the following bottleneck stage, the formation of water network on the protein surface and the formation of HBs between the protein and water also contribute to lowering the system free energy via an enthalpy decrease [22,25,30]. The protein native state residing at the bottom (the global free energy minimum region) of the funnel-like FEL (or folding funnel) is not a single, rigid conformer but exists as an ensemble of statistically populated conformational states/substates [31,32,33]. The hierarchically organized free energy wells (*i.e.*, an organization that the low-tier wells reside within the high-tier wells, and the lower tier the wells are, the lower are barrier heights between these wells [33]) determines the hierarchy of protein dynamics—different structural components feature differentiated amplitudes and timescales of the fluctuations [2]. Of particular note is that the solvent is able to influence protein dynamics at various hierarchical levels. It has been shown that the fast residue side chain rotations occurring on picosecond (ps) timescale are coupled to the hydration-shell fluctuations, and that the relatively slow loop motions occurring on nanosecond (ns) timescale are slaved to bulk-solvent motions [4,5,34,35]. Furthermore, the complete exchange of protein-bound water molecules arising from water translational motions have been proposed to be necessary for protein structural relaxation that allows anharmonic and collective motions in proteins [36]. Because the hierarchical arrangement of free energy wells determines that the higher-tier protein motions result from the lower-tier fluctuations [34,37,38], it is reasonable to postulate that the protein collective motions, which involve the entire protein structure and occur on the timescales of microsecond (μs) to millisecond (ms), are a consequence of the accumulation of loop motions and side chain rotations, in which the solvent motions play an important role [22,39,40].

The mobility of the atoms, and further, of the atomic groups and the molecules, is temperature dependent because the temperature is a fundamental determinant of the atomic thermal energy. A generally observed phenomenon is that the protein conformational flexibility increases/decreases with the elevated/decreased temperatures of the protein–solvent system. The fact that solvent fluctuations affect protein dynamics at various hierarchical levels raises a question of whether the temperature of the solvent, or that of the protein itself, is a primary factor in determining the internal motions of the protein. The second law of thermodynamics determines that the thermal energy always distributes as evenly as possible over the entire protein–solvent system, thus making it difficult to distinguish experimentally the effect of the solvent temperatures from that of the protein temperatures on protein dynamics [41]. The method of MD simulation can be a valuable alternative, since it can create a thermodynamic system with the protein at one temperature and the solvent at a different temperature [41]. Using such a simulation procedure, Vitkup *et al.* [42] have shown that, in the temperature range 180–300 K, the elevated solvent temperature, and hence the increased solvent mobility, plays an essential role in enhancing the amplitude of atomic fluctuations in the protein, but that the temperatures of the protein itself only weakly affect the amplitude of protein atomic fluctuations. Despite this pioneering work, the detailed information about effects of the solvent temperatures on the molecular motions and conformational space sampling of the protein, as well as on the FEL of the protein–solvent system, remains unclear. Moreover, the question of how the solvent mobility dictates the hierarchical dynamics, from the low-tier group fluctuations to the high-tier collective motions of the protein, remains unanswered.

In order to resolve the above issues, in the present work the proteinase K (which is a subtilisin-like serine protease from *Tritirachium album* limber with a broad-spectrum degradation capability to degrade proteins [43,44]) in explicit solvent was subject to a series of MD simulations with temperatures of the protein and the solvent coupled to either 180 or 300 K. Proteinase K was chosen because it is a medium-sized globular α/β protein (279 amino acids) with an available high-resolution X-ray crystal structure [45] and, in particular, its dynamic properties, molecular motions, and structure–dynamics–function relationship have been investigated by us in a series of simulation/modeling studies [46,47,48,49,50,51,52]. The long, multiple-replica MD simulations with different initial atomic velocities [53,54] were performed on each system of different protein/solvent temperature combinations to guarantee a more effective sampling of the conformational space accessible to proteinase K. Apart from comparative analyses of several structural properties, the differences in the sampled protein conformational spaces, collective motions, and constructed FELs between different temperature combinations were compared. The reasons for the enhanced mobility/flexibility of proteinase K caused by the elevated solvent temperature were discussed in depth based on the competitive HB interactions between the protein and solvent and between atoms within the protein. Finally, a refined FEL model was proposed to explain the role of the solvent in determining the hierarchical dynamics of proteins.

## 2. Results

### 2.1. Evaluation of the Conformational Sampling

The time evolution of backbone RMSD relative to the starting structure was computed for each replica of the four simulation systems. The results (Supplementary Figure S1) show that for the systems P180/S180 and P300/S180, proteinase K requires only a few ps to reach stable RMSD values, and for the systems P180/S300 and P300/P300, the protein requires about 600 ps and about 1 ns to reach an approximate equilibrium, respectively. In order to guarantee the consistency of our data over time among the four simulation systems, the first 1-ns trajectories were discarded and only the equilibrated portions (1–15 ns) of each replica were concatenated, thus yielding four 84-ns single joined trajectories.

The cosine contents for the first three eigenvectors derived from ED analyses of the replicas and of the joined trajectories were calculated to assess the conformational sampling. The results (Supplementary Table S1) show that, for the independent replicas of each simulation system, most of their eigenvectors have significantly higher cosine contents (close to 1) than the corresponding eigenvectors of the single joined trajectory (~0.006 to ~0.2). This suggests that the regions of conformational space sampled by these independent replicas are displaced in different directions from the starting structure, resulting in different but partially overlapping samplings. Our strategy of performing the multiple, long simulations therefore improves the ergodicity of the system and achieves a relatively higher degree of sampling convergence in the joined trajectory than in the individual replicas. Consequently, the single joined trajectories of the four simulation systems were used for further analyses except where otherwise specified.

### 2.2. Structural Properties

Table 1 lists the average values and standard deviations (in parentheses) for several structural properties of proteinase K calculated based on the joined trajectories at different combined temperatures. Under the combination of P180/S180, proteinase K has the largest average values for NNC and SSE while the smallest average values for RMSD and SASA, whereas under the P300/S300 combination, proteinase K has the smallest average values for NNC and SSE but the largest values for RMSD and SASA. This implies that the simulations at the combined temperature P180/S180 lead to the most stable structure and the most compact packing of proteinase K, whereas the opposite situations occur when simulations were performed at P300/S300. Interestingly, although the differences in structural properties between P300/S180 and P180/S300 are minor, proteinase K has a greater number of inter-atomic contacts, more compact packing, and smaller structural deviation from the starting structure when the solvent temperature is at 180 K than when at 300 K. In conjunction with larger standard deviations of all structural properties at P180/S300 than at P300/S180, these results indicate that the effect of the solvent temperature overwhelms that of the protein temperature, resulting in a higher rigidity/flexibility at the low/high solvent temperature.

When the solvent temperatures increase from 180 to 300 K while the protein temperatures remain unchanged, e.g., at 300 K (or 180 K), the NNC and number of residues in SSE decrease by 1.5% (0.9%) and 6.6% (3.3%), respectively, and the RMSD and SASA increase by 82.3% (45.2%) and 2.7% (1.8%), respectively; when the solvent temperatures remain at 300 K (or 180 K) while the protein temperatures increase from 180 to 300 K, the NNC and number of residues in SSE decrease by 1.2% (0.6%) and 5.4% (2.1%), respectively, and the RMSD and SASA increase by 37.8% (9.7%) and 1.9% (1.0%), respectively. These results reveal that, although the changes in protein temperatures have effects on structural properties of proteinase K, their effects are weaker and quantitatively smaller than those of the solvent temperature changes.

The Rg of proteinase K is 16.6 and 16.7 Å when the solvent is at 180 and 300 K, respectively, independent of the protein temperature. In addition, for all of the structural properties, their standard deviations follow the order P180/S180 < P300/S180 < P180/S300 < P300/S300, implying that proteinase K experienced larger structural variations and hence, a stronger internal mobility, during simulations at the high solvent temperatures than at the low solvent temperatures.

### 2.3. Structural Flexibility

**Figure 1:**
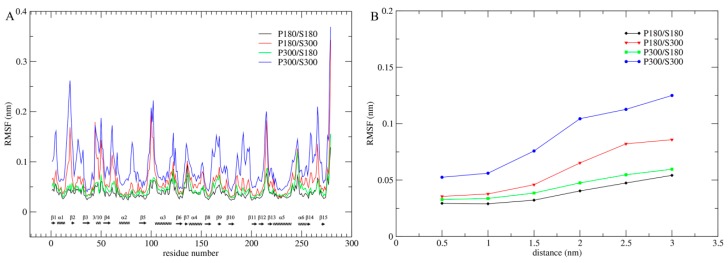
Flexibility profiles of proteinase K calculated from the joined trajectories at the four combined temperatures. (**A**) Per-residue C_α_ RMSF profiles as a function of residue number. SSEs were marked along the horizontal axis with spirals and arrows representing α (or 3/10) helices and β strands, respectively; (**B**) C_α_ RMSF values as a function of distance from the protein core to the protein surface. In both (**A**) and (**B**), the black, red, green, and blue lines are profiles corresponding to the combined temperatures P180/S180, P180/S300, P300/S180, and P300/S300, respectively.

Per-residue C_α_ root mean square fluctuation (RMSF) was computed from the joined trajectories to evaluate and compare the structural flexibility of proteinase K under the four different temperature combinations (Figure 1A). As shown in Figure 1A, although proteinase K exhibits the highest and lowest overall structural flexibility at the combined temperatures P300/S300 and P180/S180, respectively, the low (P300/S180) and high (P180/S300) solvent temperatures significantly reduce and elevate the overall protein flexibility, respectively, leading to very similar flexibility profiles between P300/S180 and P180/S180, and between P180/S300 and P300/S300. Therefore, the intensity of atomic fluctuations, and hence the protein internal flexibility, is strongly related to the solvent temperatures. Of particular note is that the solvent temperatures have a greater influence on the flexibility of loop regions than on that of the SSEs. For instance, the intensity of loop fluctuations is drastically increased and decreased when the temperature combination goes from P180/S180 to P180/S300 and from P300/S300 to P300/S180, respectively, whereas the corresponding changes in the intensity of SSE fluctuations are smaller, implying that the solvent-temperature-dependent changes in protein flexibility originate from the loop motions.

In order to further determine the effect of the solvent temperatures on flexibility of the entire protein structure, we computed the C_α_ RMSF values as a function of distance to the protein surface (Figure 1B). As shown in Figure 1B, whether at the protein surface or in the protein core, the RMSF values are in an order P300/S300 > P180/S300 > P300/S180 > P180/S180 and, for these four combinations, a consistent trend is observed: RMSF values increase with increased distance from the protein core to the surface. However, the degree of this increase is different, with the most dramatic increase observed for P300/S300, large increase for P180/S300, and small increase for P300/S180 and P180/S180, ultimately leading to the amplitudes of atomic fluctuations, which are distributed over the entire protein structure, in the order P300/S300 > P180/S300 > P300/S180 > P180/S180.

For all four temperature combinations, the largest amplitudes of atomic fluctuations occur invariably on the protein surface. This is mainly due to: (i) the lack of structural packing environment for the surface-exposed atoms; and (ii) the direct interactions/contacts between the protein surface atoms and the water molecules. Nevertheless, the ordinal increase in RMSF values of the protein core from the low to the high solvent temperatures indicates that the mobility of the solvent can also determine the amplitude of atomic fluctuations in the protein core. The small increases in RMSF values from P180/S180 to P300/S180 reveal that elevating the temperature of the protein does make a contribution, to some extent, to its own mobility. Moreover, the degrees of these RMSF increases are relatively consistent over the entire protein structure (from the core to the surface), which may reflect a pure thermal relaxation of the protein arising from the elevated protein temperature. When compared to P300/S180, under the P180/S300 combination the protein shows not only significantly higher surface and near-surface RMSF values but also slightly higher core RMSF value. This result reveals that the high surface mobility caused by the high solvent temperature is not only transmitted to the protein core, but also improves the mobility of protein core.

### 2.4. ED and Collective Motions

In order to investigate the effect of the solvent temperatures on the collective motions (or global large-scale concerted motions) of proteinase K, ED analyses were performed on protein C_α_ atoms using the joined trajectories of the four simulation systems.

The total mean square fluctuation (TMSF) values obtained by diagonalization of the covariance matrices are 2.97, 1.33, 0.64, and 0.47 nm^2^ for the combinations of P300/S300, P180/S300, P300/S180, and P180/S180, respectively, indicating that proteinase K experiences significantly larger atomic fluctuations when the solvent temperatures are at 300 K than when at 180 K, in agreement with the above structural flexibility analysis. When the temperature combination goes from P300/S180 to P300/S300 and from P180/S180 to P180/S300, the TMSF values increase by 3.64- and 1.83-fold, respectively; the TMSF values increase by 1.23- and 0.36-fold when the temperature combination goes from P180/S300 to P300/S300 and from P180/S180 to P300/S180, respectively. These results reveal that elevating the temperature of the solvent makes a substantial contribution to increasing protein fluctuations. Although elevating protein temperature also contributes to protein fluctuations, such a contribution is quantitatively smaller, especially when the solvent temperature is at 180 K.

**Figure 2:**
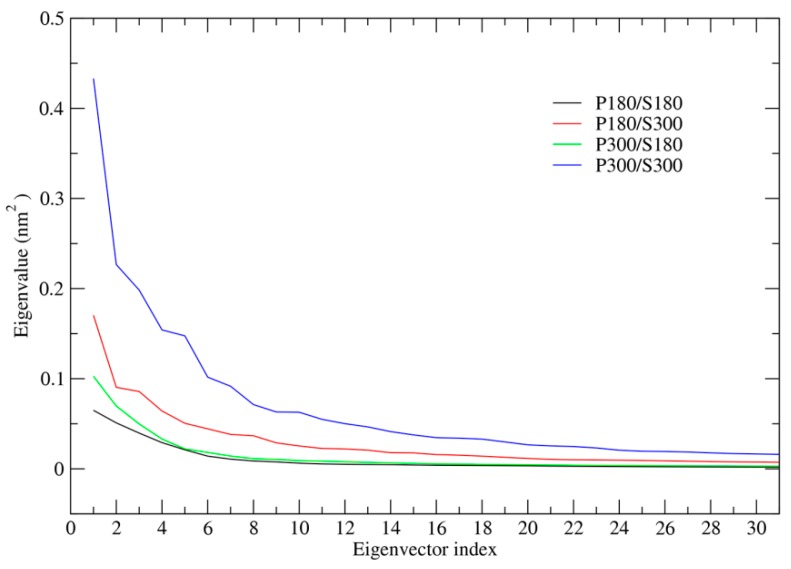
Eigenvalues as a function of eigenvector index derived from ED analyses of the joined trajectories at the four combined temperatures. Only eigenvalues of the first 30 eigenvectors (total is 837) are shown. Black line: P180/S180; red line: P180/S300; green line: P300/S180; blue line: P300/S300.

Figure 2 shows the eigenvalues as a function of eigenvector index. The four simulation systems share a common trend: the largest eigenvalue is for the first eigenvector and then the eigenvalues decrease with increased eigenvector index until they reach a minimum plateau. However, the degree of the steepness for these four eigenvalue curves is different and follows an order P300/S300 > P180/S300 > P300/S180 > P180/S180. Apparently, the first few eigenvectors of P300/S300 have the highest eigenvalues among those of the four combinations. The second highest, low, and the lowest eigenvalues of the first few eigenvectors are observed for the P180/S300, P300/S180 and P180/S180, respectively. These results indicate that the high solvent temperatures enhance greatly the anharmonic atomic motions and thus promote the global collective motions of the protein along the first few eigenvectors. In contrast, at the low solvent temperatures (*i.e.*, P300/S180 and P180/S180), the first few eigenvectors have eigenvalues only slightly higher than those of eigenvectors with higher index, implying that the insignificant anharmonic atomic motions predominate along the first few eigenvectors, and as thus, the collective motions of the protein are greatly suppressed.

**Figure 3:**
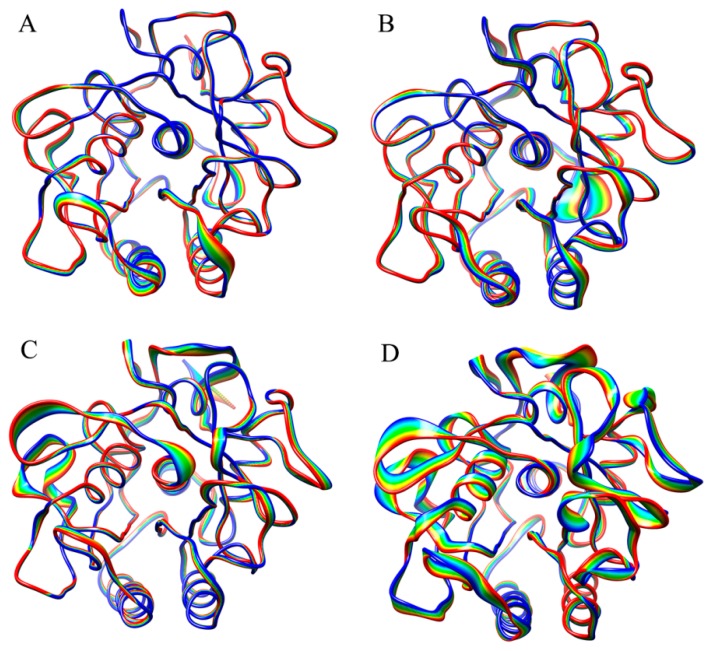
Projection extremes of the first eigenvector obtained from ED analyses of the joined trajectories at the four combined temperatures: (**A**) P180/S180; (**B**) P300/S180; (**C**) P180/S300; and (**D**) P300/S300. The linear interpolations between the two extremes are colored from blue to red to highlight conformational differences between these two states but do not represent the transition pathway.

Figure 3 shows the projection extremes of the first eigenvector, which represent the most significant collective motions of proteinase K at each combined temperature. It should be noted that the linear interpolations between these two extremes are not the conformational transition pathway between the two extremes but emphasize merely the conformational differences between them. Under the P300/S300 combination (Figure 3D), proteinase K exhibits the most significant collective motions that involve conformational displacements over the entire structure, with the large and small displacements observed in the surface-exposed loops and SSEs, respectively. The second significant collective motions of proteinase K are observed for the P180/S300 combination (Figure 3C), which involve conformational displacements in most of the surface loops and some of the SSEs. Under the P300/S180 (Figure 3B) and P180/S180 (Figure 3A) combinations, most of the structural regions in the protein do not exhibit apparent displacements and only a very limited number of surface-exposed loops exhibit the large displacements. These observations are consistent with the eigenvalue order of the first eigenvector described above, both suggesting that the high solvent temperatures promote the global collective motions of the protein.

**Figure 4:**
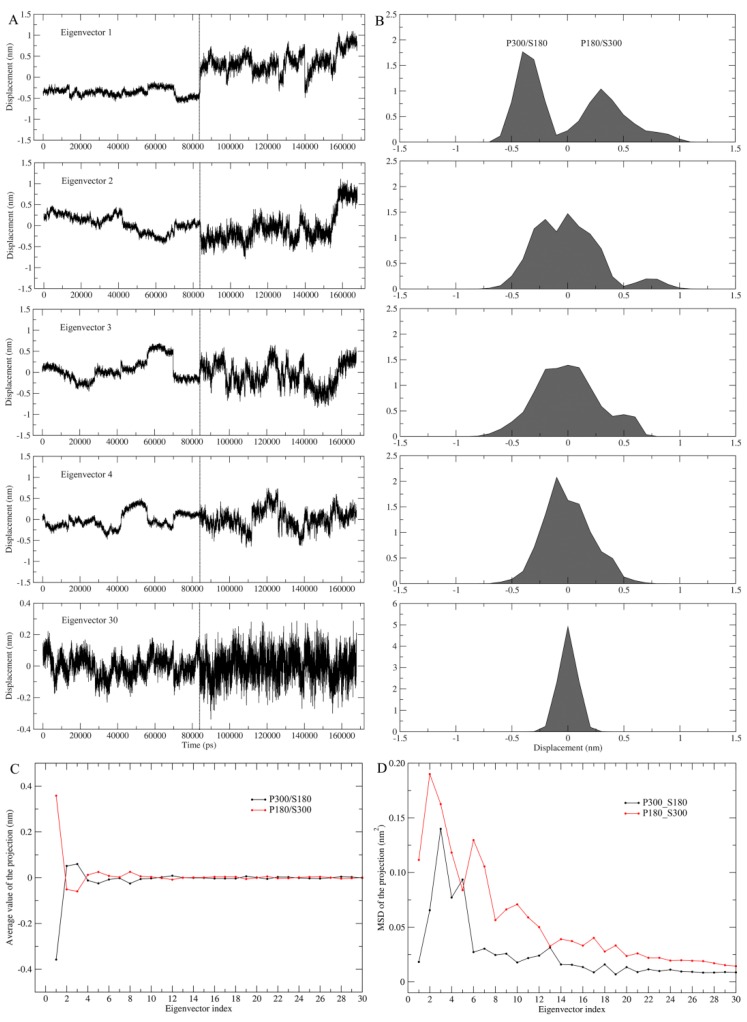
Eigenvector projections and their properties obtained from combined ED analysis of the merged MD trajectories of P300/S180 and P180/S300. (**A**) Projections of the merged trajectory (P300/S180: 0–84 ns; P180/S300: 84–168 ns) onto the combined eigenvectors 1–4 and 30; (**B**) Distributions of the corresponding eigenvector projections; (**C**) Average values of the first 30 eigenvector projections as a function of eigenvector index; (**D**) Mean square displacement (MSD) values of the first 30 eigenvector projections as a function of eigenvector index. The projection properties (average and MSD values) were calculated separately from the two equal halves of a combined eigenvector projection that correspond to the P300/S180 and P180/S300 parts, respectively.

Combined ED analyses were performed to investigate the differences in ED properties. There are six possible pairwise combinations among the four joined trajectories; for purpose of clarity, we choose only the pair of P300/S180–P180/S300 to demonstrate the effect of the solvent temperatures on ED properties. Figure 4 shows the projections of the merged trajectories onto the combined eigenvectors and the properties of these projections. Only in the case of the first combined eigenvector can the projection be well separated (top panel, Figure 4A), which is reflected clearly by the distinctly different projection distributions (top panel, Figure 4B) and average values (Figure 4C) between P180/S300 and P300/S180. This reveals that the changes in the protein and solvent temperatures lead to different equilibrium fluctuations and average structures of proteinase K along the first combined eigenvector. Starting from the combined eigenvector 2, the two equal halves of the projection show gradually increasing overlap between their distributions, as well as increasing similarity between the average values, e.g., when reaching the eigenvector 30, the projection distributions overlap into a near-Gaussian distribution and the average values are almost identical. These results indicate a gradually increasing similarity between the motion modes and average structures of the protein under these two temperature combinations, implying that the degree of harmonicity in protein motions increases with increased eigenvector index.

Of particular note is that for each of the first few combined eigenvectors, the right half of the projection (corresponding to P180/S300) exhibits a larger amplitude of the fluctuations than the left half of the projection (corresponding to P300/S180) (Figure 4A). This reflects that at the high solvent temperature, proteinase K experiences more dramatic conformational changes along these combined eigenvectors than at the low solvent temperature. Figure 4D shows the MSD values for the two halves of the projection as a function of eigenvector index, which can be used to quantitatively compare the difference in degree of the protein conformational shifts at different combined temperatures. Apparently, most of the eigenvectors (with the exception of eigenvectors 5 and 13) have significantly larger MSD values when the combined temperature is at P180/S300 than when at P300/S180, revealing that the high solvent temperature greatly facilitates the conformational shifts/changes of the protein in a subspace spanned by these eigenvectors.

### 2.5. Sampled Conformational Subspaces

**Figure 5:**
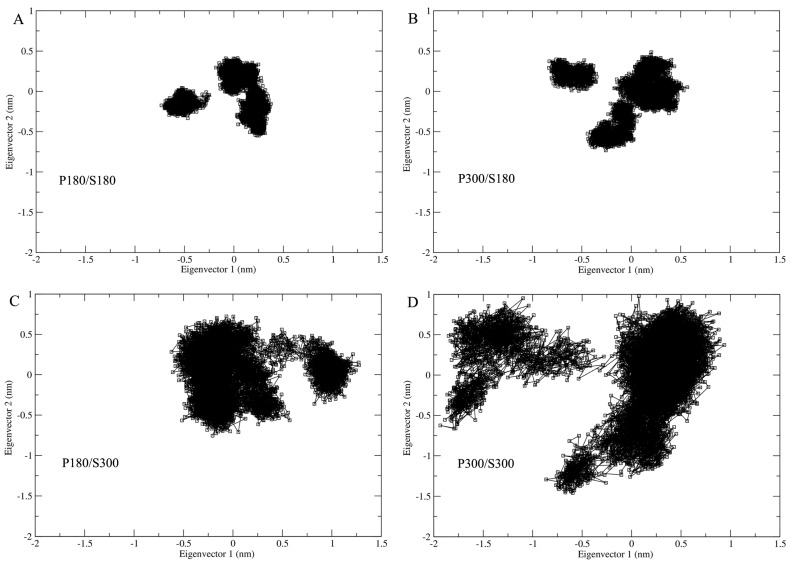
Projections of the joined MD trajectories onto the two-dimensional conformational subspace spanned by the first two eigenvectors: (**A**) P180/S180; (**B**) P300/S180; (**C**) P180/S300; and (**D**) P300/S300.

In order to examine the influence of the solvent temperatures on the extent of conformational space explored by proteinase K, the four joined trajectories were projected onto a two-dimensional conformational subspace spanned by the first two eigenvectors (Figure 5). As shown in Figure 5, proteinase K explores similar shapes of the sampled regions under the temperature combinations P180/S180, P300/S180, and P300/S300, with the smallest and largest regions observed for P180/S180 and P300/S300, respectively. The second largest region is observed for the P180/S300; although this region exhibits a different shape with respect to the other three ones, it not only covers the entire regions of P180/S180 and P300/S180, but also has the largest overlap with that of P300/S300, revealing that the simulations at the high solvent temperatures enlarge the sampled conformation space of the protein.

As described above, elevating solvent temperature facilitates not only greatly the protein surface atomic fluctuations but also to a certain extent the fluctuations of atoms in the protein core (Figure 1B). In order to further determine the influence of the solvent temperatures on the sampling degrees of the protein core, core periphery, and surface loops, these three structural parts were projected onto the subspace spanned by the first two eigenvectors (Supplementary Figure S2). Visualization of the sampled conformational spaces reveals that: (i) for all four temperature combinations, the protein surface loops, core periphery, and protein core sample the largest, moderate, and smallest regions, respectively; (ii) the surface loops sample significantly more extended regions at the high solvent temperatures (P180/S300 and P300/S300) than at the low solvent temperatures (P300/S180 and P180/S180); and (iii) the core periphery and protein core explore relatively larger regions at the high solvent temperatures than at the low solvent temperatures. The above results reveal that the increased solvent temperatures facilitate not only the conformational sampling of the surface loops but also those of the structural parts that have no direct contacts/interactions with the solvent, in agreement with the comparative analysis of RMSF values as a function of distance to the protein surface.

### 2.6. Free Energy Landscapes

**Figure 6:**
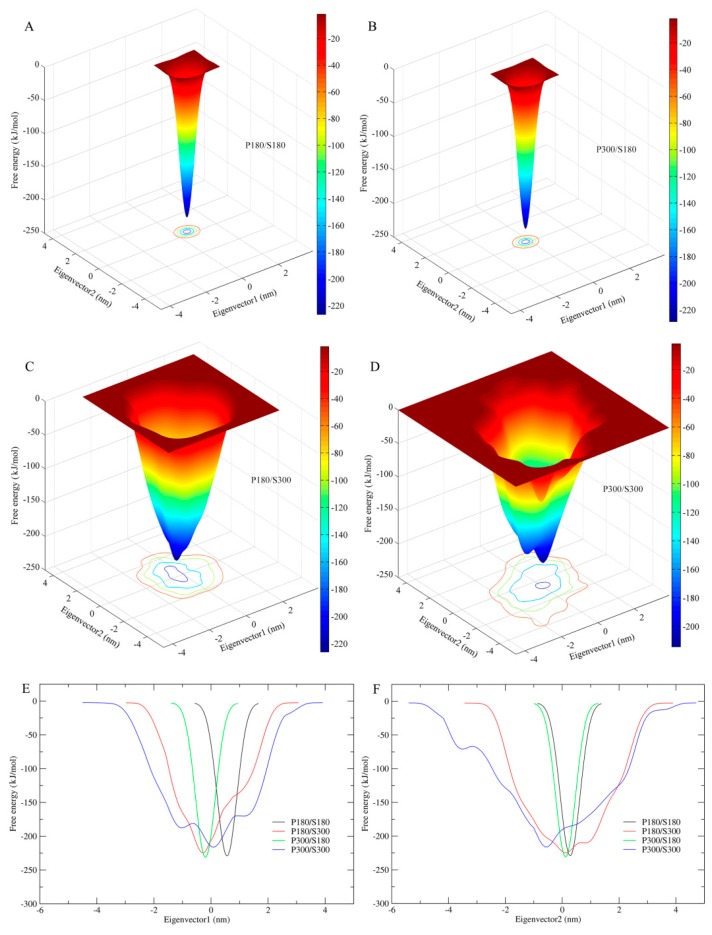
Constructed FELs of the proteinase K-solvent system at the four combined temperatures. (**A**–**D**) FELs as a function of two-dimensional conformational subspace at the combined temperatures: (**A**) P180/S180; (**B**) P300/S180; (**C**) P180/S300; and (**D**) P300/S300. The color bar represents the free energy value in unit of kJ/mol; (**E**,**F**) One-dimensional FELs along the projection of the eigenvectors (**E**) 1 and (**F**) 2. Black line: P180/S180; red line: P180/S300; green line: P300/S180; blue line: P300/S300.

Figure 6 shows the FELs of the protein–solvent systems at the four combined temperatures as a function of two-dimensional conformational subspace (Figure 6A–D) and of one-dimensional eigenvector projection (Figure 6E,F). It should be pointed out that: (i) the present ns-timescale MD simulations sample only a limited portion of the protein accessible conformational space (e.g., the conformational space close to the native state but containing no the unfolded states); (ii) despite the reduction of dimensionality to the two- or one-dimensional space, protein conformational space is highly multi-dimensional; and (iii) the eigenvector projection biases towards second-order correlations of protein motions that result in conformational populations with mixed properties [55]. Therefore, the eigenvector-projection-based FELs constructed from the metadynamics simulations are incomplete, representing only a major portion of the landscape but containing no detailed characteristics of the free energy surface (e.g., multiple minima or hierarchical arrangement of the free energy wells). Nevertheless, comparison between these FELs is still useful in probing characteristic differences resulting from alternation of the solvent/protein temperatures because the differences in statistical trends are still preserved.

As shown in Figure 6A–D, all four FELs present a funnel-like shape. However, it is apparent that the FELs of the low solvent temperatures (P180/S180 and P300/S180) exhibit a perfectly smooth surface, whereas the surfaces of P180/S300 and P300/S300 feature a weak and strong rough character, respectively. This implies that the high solvent temperatures are able to produce local free energy minima either on the funnel wall or at the funnel bottom, thus increasing the protein conformational diversity or the number of conformational states/substates near the native state and hence the flexibility of the protein. The width of the FELs, from the bottom to the top outer surface, is found to be narrowest for P180/S180 and P300/S180, whereas for P180/S300 and P300/S300, the funnel width is obviously wide and the widest, respectively (Figure 6E,F). This reveals that the high solvent temperatures greatly increase the conformational entropy of the protein, whereas the protein temperatures have only minor effect on the protein conformational entropy. Another interesting result is that the FEL corresponding to the P300/S300 has a pronounced higher minimum free energy level than the other three FELs. Although the other three FELs have similar minimum free energy values, the value of the P180/S300 is slightly higher than those of P180/S180 and P300/S180. This suggests that proteinase K has lower thermostability (or structural stability) at the high solvent temperatures than at the low solvent temperatures.

### 2.7. HB Interactions

HBs are the most important class of non-covalent interactions/forces that can contribute not only to the stability [56,57] but also to the flexibility [58,59] of the protein. In order to ascertain the origin of the characteristic differences in FELs under the four temperature combinations, we calculated the HB properties for both static and dynamic HBs within the protein and between the protein and solvent based on the joined MD trajectories.

Table 2 lists the properties of the static and dynamic HBs formed inside the protein. The static HB number, which refers to that averaged over all frames in a trajectory, is 239.2 ± 5.3, 232.9 ± 6.1, 229.0 ± 6.8, and 215.6 ± 7.8 for P180/S180, P300/S180, P180/S300, and P300/S300, respectively. A clear trend is that at the low solvent temperatures the protein has higher numbers of internal HBs than at the high solvent temperatures, although lowering the protein temperature (when the solvent temperatures remain unchanged) can still increase the HB number, e.g., 6.3 from P300/S180 to P180/S180 and 13.4 from P300/S300 to P180/S300. The static HBs play a crucial role in stabilizing protein structure because the HB formation is an exothermic electrostatic interaction that contributes to lowering the system free energy via a negative enthalpy change. In this context, the static HB number can be considered as indicative of the structural stability/rigidity. Therefore, the above results show that the low solvent temperatures increase the protein structural stability/rigidity through increasing the static HB number. Interestingly, the number of the dynamic HBs, which refers to the total number of HBs that have ever appeared in a MD trajectory, exhibits an opposite trend with respect to that of the static HBs. As shown in Table 2, significantly higher dynamic HB numbers occur at the high solvent temperatures (P180/S300 and P300/S300) than at the low solvent temperatures (P180/S180 and P300/S180), indicating that elevating the solvent temperature increases the number of the dynamic HBs inside the protein. The dynamic HB number is inversely correlated with the average HB persistency: the higher the number of the dynamic HBs, the lower is the average value of the bond persistency, and *vice versa* (Table 2); this implies that it is the increase of the low-persistence HBs that leads to an increase in the dynamic HB number. A low-persistence HB forms transiently and is easy to break during a simulation so that its donor and/or acceptor can form new HBs with the neighboring donor and/or acceptor atoms, thus leading to competitive HB interactions and, ultimately, the increase in the number of the dynamic HBs. Because the competitive interaction is able to cause conformational transition, the dynamic HB number can be considered as indicative of the structural flexibility or even as an approximate measure of the protein conformational entropy. To this end, the high solvent temperatures increase the protein flexibility/mobility through increasing the dynamic HB number.

Protein internal HBs involve either the main chains or the side chains of amino acid residues and therefore can be divided into three classes: the main chain–main chain, main chain–side chain, and side chain–side chain HBs. Although elevating the solvent temperature increases the numbers of all these three classes of HBs, the bonds involving the side chains (including main chain–side chain and side chain–side chain HBs) make a greater contribution than do the main chain–main chain HBs to the increase in the dynamic HB number (Table 2). This is not surprising, because the side chains have a larger degree of freedom than the main chains and as thus are more sensitive to changes of the solvent temperature.

Table 3 lists the properties of the static and dynamic HBs formed between the protein and the solvent, which directly reflect the changes in protein–solvent interactions among the four temperature combinations. The high solvent temperatures (P180/S300 and P300/S300) result in the lower/higher numbers of the static/dynamic protein–solvent HBs than do the low solvent temperatures (P180/S180 and P300/S180), resembling the situations of the protein internal HBs. The increased static protein–solvent HB interactions at the low solvent temperatures suggest the role of the low-temperature water molecules in rigidifying/freezing the protein structure. Because of the significantly enhanced mobility of the water molecules when the solvent temperatures go from 180 to 300 K, the numbers of the dynamic HBs at the high solvent temperatures are at least two orders of magnitude higher than those at the low solvent temperatures (Table 3). This is also reflected by the two orders of magnitude reduced HB persistency from the low- to the high-solvent temperatures, suggesting that it is the high-frequency competitive interactions between different water molecules and the protein atoms that give rise to the increased dynamic HB number and thus, the increased mobility of the protein. For all four temperature combinations, the HBs formed between the amino acid side chains and the water molecules (side chain-solvent HBs) account for ~90% of the corresponding dynamic HBs and exhibit a slightly lower persistency than the main chain–solvent HBs, revealing that the side chains are the primary participant of the protein–solvent HBs or, in other words, water molecules affect the protein dynamics mainly through their interactions with the amino acid side chains.

## 3. Discussion

“Glass transition” (or dynamical transition) of proteins, like that of glass-forming liquids [60,61], is a well-established phenomenon for all proteins manifesting as a dramatic change in their dynamical properties at approximately 200 K [17,18,19,42,62], *i.e.*, at temperatures above 200 K, the protein dynamic behavior is dominated by the large-scale collective motions involving the entire protein structure, while at temperatures below 200 K, the simple harmonic atomic vibrations predominate, thus resulting in the cease of the protein activity. Because the dried proteins do not show any dynamical transition [36,63,64] and the glass transitions in the protein and its hydration water occur at roughly the same temperature [17,65], the protein glass transition is believed to be induced by the glass transition of the solvent. In order to provide detailed information about the effect of the solvent temperatures on the changes in dynamics of the protein and, further, to probe the mechanism by which the solvent mobility dictates the hierarchical dynamics of the protein, we take advantage of the molecular simulation technique in which the protein and solvent temperatures are coupled to either 300 K (above the protein glass transition temperature) or 180 K (below the transition temperature). In contrast to the previous MD study performed on a relatively small protein carboxy-myoglobin (153 amino acids) with a short production simulation time (only 100 ps) [42], our simulations were performed on a relatively large globular protein (279 amino acids), the serine protease proteinase K, with application of the long, multi-replica simulation strategy to ensure a more effective and extensive conformational sampling of the protein. Indeed, the evaluation of cosine contents for the first few eigenvectors shows that the single joined trajectories from the multiple simulations achieve a higher degree of sampling convergence than the individual replicas, and therefore our comparative analyses based on these joined trajectories can reflect differences in intrinsic dynamic properties of proteinase K caused by different protein/solvent temperature combinations.

As a necessary complement to this work, we have also evaluated the influence of protein temperatures on the solvent mobility by calculating the mean square displacement (MSD) of the solvent in the P300/S180 and P180/S300 trajectories. The results (Supplementary Figure S3) show that under the P180/S300 combination, the water molecules demonstrate a free diffusion behavior, and thus, are not “frozen” by the low protein temperature, whereas under the P300/S180 combination the MSDs of the hydration water and bulk water are characterized by an initial rise followed by a plateau formation, reflecting that the water molecules are in a structurally arrested, glass-like state. Of interest is that in the P300/S180 system the hydration water molecules are found to have slightly higher MSD values than the bulk water during both the rising and plateau stages. This indicates that although the hydration water molecules remain still localized or arrested around the protein surface (*i.e.*, they are not “melted” by the high temperature of the protein), the high temperature of the protein can improve, to a certain extent, their diffusivity due to the physical contacts between the protein and the hydrated water molecules. Taken together, it can be concluded that the protein temperatures have only a very limited effect on the diffusivity (mobility) of the solvent.

The comparative analyses of several static structural properties and of conformational flexibility reveal that the solvent temperatures have an overwhelming effect, when compared to the protein temperatures, on these properties and atomic fluctuations of proteinase K. For instance, the changes of the solvent temperatures can lead to significant changes in average structural properties investigated (Table 1), C_α_ RMSF profiles (Figure 1A), and TMSF values, whereas the changes of the protein temperatures only result in minor changes in the structural properties and flexibility of proteinase K. Our results are consistent with previous MD simulation on myoglobin [42], both revealing the dominant role of the solvent temperature in determining the internal mobility and flexibility/rigidity of the protein.

The ED analyses reveal that at the high solvent temperatures, the motion mode described by the first eigenvector is the global collective motions involving displacements of the entire protein structure. On the contrary, at the low solvent temperatures, the large displacements were observed only in very limited structural regions, indicating that the low solvent temperatures greatly suppress the collective motions of proteinase K. Further combined ED analyses reveal that the dramatic conformational shifts occur only at the high solvent temperatures, while at the low solvent temperatures, the simple harmonic fluctuations predominate even along the first few eigenvectors. In our previous MD studies [46,47,48,49], the collective motions of proteinase K have been shown to not only lead to the breathing/twisting of the substrate-binding channel and pockets, but also play a role in modulating dynamic behaviors of the structural regions not directly involved in the catalysis but indirectly influencing the enzymatic function. Thus, the global collective motions of proteinase K are related to the substrate binding, orientation, catalysis, product release, and the regulation of these processes. The disappearance of the global collective motions below glass transition temperature explains why the proteins will cease to be active upon the glass transition.

The comparison between RMSF values as a function of distance to the protein surface, in conjunction with the comparison between sampling degrees of the conformational space explored by the protein core, core periphery, and surface loops at the four combined temperatures, reveals that: (i) the surface mobility can transmit to the core periphery and further to the core; and (ii) the mobility and sampling degrees of the non-surface-exposed regions are proportional to those of the protein surface. In light of the above findings, we can propose the following rough scenario: the high mobility of the water molecules, which arises from the high solvent temperatures, can lead to more frequent collisions/interactions with protein surface when compared to water molecules at the low temperatures, and therefore results in larger atomic fluctuations and structural displacements of the protein surface, especially of the solvent-exposed loop regions that lack the structural constraints. Such a high mobility can further transmit via specific mechanic mechanism to the protein core periphery and finally to the protein core, thus causing the global collective motions of the protein.

The number of dynamic HBs can be considered as a representative of competitive interactions within the protein and between the protein and the solvent because of the universal distribution of HBs in the protein–solvent system. Comparison between protein–solvent HB numbers reveals that the high solvent temperatures result in more intense competitive interactions between the water molecules and the protein surface atoms than do the low solvent temperatures. In addition, elevating solvent temperature also facilitates greatly the competitive interactions between atoms within the protein. In a previous theoretical study, Agarwal has investigated the transfer of energy from the solvent to the protein by adding kinetic energy to solvent molecules in the hydration shell, and the results show that within a short time, the solvent kinetic energy is transferred to the protein residues even more than 8 Å away from the protein surface, resulting in increased mobility of the protein [35]. Taken together, it is reasonable to conclude that it is the competitive interactions that transmit solvent kinetic energy/mobility to the protein surface and further to the protein interior, ultimately leading to an overall increased conformational flexibility and the collective motions of the protein.

**Figure 7:**
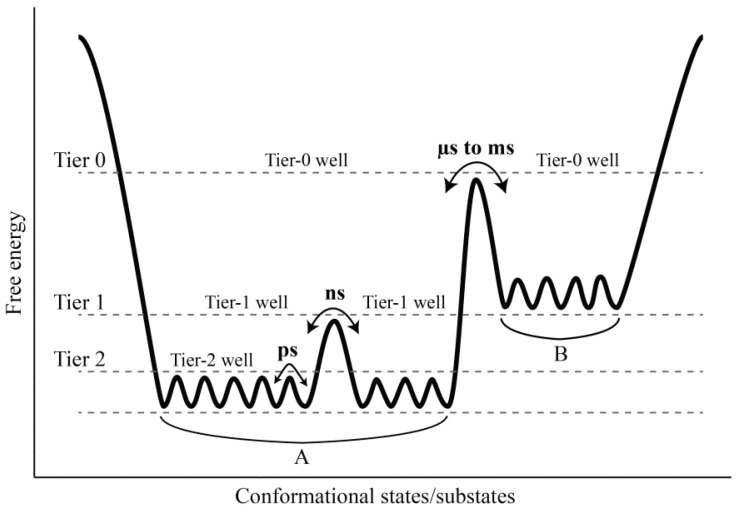
A proposed FEL model to explain the effect of the solvent mobility on protein dynamics. This model is represented as a hierarchical organization of free energy wells (*i.e.*, the smallest tier-2 wells are within the relatively large tier-1 wells, and the tier-1 wells are within the largest tier-0 wells), which dictates the hierarchical dynamics of the protein (*i.e.*, different structural components feature different amplitude and timescale of the fluctuations). The tier-2 and tier-1 substates and tier-0 states (**A** and **B**) are located within respective free energy wells. The tier-2, tier-1, and tier-0 dynamics, which are defined as conformational interconversion between respective substates/states, involve the side chain rotations on ps timescale, loop motions on ns timescale, and collective motions of the entire structure on timescale of μs to ms, respectively. The tire-0 dynamics are a result of the accumulation of the tier-1 and tier-2 dynamics. By exacerbating the competitive interactions between the protein and solvent and between atoms within the protein, the solvent mobility plays its role in facilitating the cascade amplification of microscopic motions of atoms and atomic groups into the global collective motions of the protein (for details, see the text).

Comparison between our constructed FELs reveals that elevating the solvent temperature increases the conformational substates and conformational entropy while reducing the thermostabilility of the protein. These results, in conjunction with the data presented in this work and available from the literature [2,4,5,33,34,66], allow us to propose a more refined FEL model to explain the effect of the solvent temperatures on the protein dynamics (Figure 7). It should be point out that: (i) the FEL model plotted in Figure 7 is not original, but is mainly based on the model documented in references [2] and [66]; (ii) the information about the conformational transitions occurring on timescales of μs to ms comes from the experimental data rather than the MD data presented in this work; and (iii) the model presented here is to highlight the role of the solvent mobility in determining the hierarchical dynamics of proteins as described follows. First, the elevated solvent temperature increases the kinetic energy of water molecules and hence their mobility. This will result in the high-frequency collisions/competitive interactions of the water molecules with the protein surface (especially with the surface-exposed amino acid side chains) and thus cause the rotational motions of the side chains. As shown in Figure 7, side chain rotations correspond to the tier-2 dynamics, which occur on the timescale of ps and involve crossing among the tier-2 free energy wells. Second, the accumulation of side chain rotations will break the local non-covalent interactions involved in the loop regions that lack the structural constraints, which in turn exacerbates the competitive interactions between residues and water molecules and as thus leads to the loop motions on the protein surface (*i.e.*, the ns-timescale tier-1 dynamics that involve crossing among the tier-1 free energy wells). Finally, by perturbing the water network (hydration shell) around the protein surface [6,67,68] and increasing interactions of the loops with the other protein structural parts [35], the large loop fluctuations will facilitate the competitive interactions between atoms within the protein, thus transmitting the solvent kinetic energy over the entire protein structure and leading to the global collective motions of the protein (*i.e.*, the tier-0 dynamics that occur on the timescales of μs to ms and involve crossing among the tier-0 free energy wells). Therefore, the high mobility of the solvent, which arises from its high temperature, plays a crucial role in facilitating the cascade amplification of microscopic motions of atoms and atomic groups into the protein global collective motions. However, the low solvent mobility, which results from its low temperature, will inhibit such cascade amplification through suppressing the competitive interactions between atoms within the protein and between the protein and solvent, ultimately leading to insignificant anharmonic motions of the protein as observed in this study.

## 4. Materials and Methods

### 4.1. Simulation System

The high-resolution X-ray crystal structure of proteinase K (0.98 Å, PDB ID: 1IC6) [45] obtained from Protein Data Bank (www.pdb.org) was used as an initial structure for MD simulations. The hetero atoms and crystallographic water molecules were removed while the protein atoms and the two bound Ca^2+^ cations were retained. The treated structure was soaked in a periodic cubic box filled with the single point charge (SPC) [69] water molecules, with the minimum distance of solute-box edges set to 1.4 nm. 53 chloride ions and 48 sodium ions were introduced, respectively, to the protein–solvent system by replacing the corresponding numbers of water molecules to obtain a 150-mM salt concentration while neutralizing the overall charge of the system, ultimately leading to a total of 51,926 atoms in the system.

### 4.2. MD Simulations

All MD simulations were performed using the GROMACS software package [70] with the GROMOS96 43a1 force field implemented on a parallel architecture.

Initially, the system was subject to energy minimizations (1000 steps of steepest descent followed by 1000 steps of conjugate gradient method) until no significant energy change could be detected. The molecular mechanics optimization was followed by four successive 200-ps position-restrained dynamics runs at 300 K with a gradual decrease in harmonic positional restraint force constant on the solute (Kposres = 1000, 100, 10 and 0 kJ·mol^−1^·nm^−2^). In the production MD runs, the solute (protein and Ca^2+^) and the solvent (water and counterions) were separately coupled to the v-rescale thermosat [71] at either 180 K or 300 K with a common coupling constant of 0.1 ps, thus resulting in four simulation systems of different temperature combinations: P300/S300, P180/S300, P300/S180, and P180/S180 (here the “P” and “S” refer to the protein and solvent, respectively). In order to improve the efficiency of conformational sampling, six independent 15-ns production MD simulations were performed for each system, initializing MD runs with different initial atomic velocities taken from a Maxwell distribution of the corresponding temperature. The obtained MD trajectories for the same system but characterized by different initial velocities are referred to as replica 1 to replica 6.

The other simulation protocols used are: the pressure was maintained by weakly coupling the system to an external pressure bath at one atm with a coupling constant of 0.5 ps; the LINCS algorithm [72] with order 4 was used to constrain the heavy atom bond lengths to their equilibrium positions, allowing an integration time step of 2 fs; long-range electrostatic interactions were treated using Particle-mesh Ewald (PME) summation method [73] with interpolation order of 4, Fourier grid spacing of 0.135 nm, and Coulomb radius of 1.0 nm; van der Waals (VDW) interactions were truncated at a cut-off of 1.4 nm; center-of-mass motion was removed every 1 time step; non-bonded pair list was updated every 10 steps; the structural frames were saved every 10 ps.

### 4.3. Trajectory Stability and Sampling Convergence

Trajectory stability was evaluated by computing the time-dependent backbone root mean square deviation (RMSD) relative to the starting structure. For each system of the temperature combinations, the equilibrated parts of each replica were concatenated together to obtain a single joined trajectory, representative of different sampling directions around the starting structure.

The degree of the convergence of conformational sampling was evaluated by computing the cosine contents of the first few eigenvectors obtained from essential dynamics (see below for details of ED) analyses of the MD trajectories, including replicas 1–6 and the single joined trajectories at each combined temperature. Since the first few eigenvectors represent the most significant motions of the protein, their cosine similarity is a good indicator to assess whether the conformational sampling is converged or not [74]. If the first few eigenvectors have a high cosine content (*i.e.*, close to 1), the corresponding largest scale motions in the protein dynamics resemble random diffusion, and thus the sampling is insufficient on the timescale of the simulation [54]. In contrast, the value close to 0 means a converged sampling.

### 4.4. Structural Properties

The static structural properties of each frame extracted from the joined equilibrium trajectories, including the solvent accessible surface area (SASA), number of native contacts (NNC), radius of gyration (Rg), backbone RMSD with respect to the starting structure, and number of residues in secondary structural elements (SSE) were calculated using the programs g_sas, g_mindist, g_gyrate, g_rms, and do_dssp [75] within GROMACS, respectively. These properties were then averaged over all the frames in a trajectory.

### 4.5. Properties of HBs

The protein internal HBs and the protein–solvent HBs were detected using the program g_hbond within GROMACS and the Hydrogen Bonds plugin within VMD [76], respectively. The geometric criteria chosen for a HB are: minimum angle of 120° for donor–hydrogen–acceptor and maximum donor–acceptor distance of 3.5 Å. The HB persistency, which is used as an index of the bond stability, is defined as the fraction of the frames in which a specified bond exists out of the total frames contained in a trajectory. The HBs that exist in an extracted structural frame are defined as the static HBs; the corresponding HB number is referred to as the static HB number. The HBs that have ever existed in a trajectory are defined as the dynamic HBs; the corresponding number is referred to as the dynamic HB number. The numbers of HBs, including the dynamic and static, were counted for both the protein internal HBs and protein–solvent HBs.

### 4.6. Essential Dynamics (ED)

ED [77,78], which is equivalent to the principal component analysis (PCA) in mathematics, is a powerful tool for reducing the high-dimensional data down to the low-dimensional aspects that reveal the large-scale concerted motions (or collective motions) of the protein. This method is based on a diagonalization of the covariance matrix built from atomic fluctuations in a trajectory, yielding a set of the eigenvectors and corresponding eigenvalues. The eigenvectors are directions in the conformational space and represent the collective displacements of groups of atoms along those directions; the eigenvalues are atomic mean square fluctuations (MSF) of the corresponding eigenvectors. Generally the first few eigenvectors constitute an essential conformational subspace within which the most significant large-scale concerted motions take place. The covariance matrices of C_α_ atoms were built and diagonalized using the program g_covar within GROMACS; the projections of trajectories onto the eigenvectors were performed using the g_anaeig program within GROMACS.

A useful method for comparing the ED properties of two simulations is the combined ED [79]. In this method, ED is performed on a combined trajectory concatenated from different simulations. The differences in projection properties (*i.e.*, the distribution, average value, and mean square displacement (MSD) of the projection) between different parts of the combined eigenvector can provide information on differences in equilibrium fluctuations (or average structures) and in conformational shifts between different simulations.

### 4.7. Metadynamics and FEL

The Metadynamics [80,81] is a powerful tool for both accelerating rare event sampling and reconstructing FEL by introducing a history-dependent bias potential acting on a restricted number of collective variables (CVs). The well-tempered metadynamics [82] is an improved version that solves the convergence problem of the standard metadynamics through decreasing the bias potential growth rate. Therefore, the well-tempered metadynamics simulations were performed to reconstruct the FELs of the systems under the four different protein/solvent temperature combinations.

The CV1 and CV2 used in metadynamics simulations were projection of MD trajectory onto the eigenvectors 1 and 2, respectively. The starting structure was the final snapshot from the standard MD simulation. The initial Gaussian height was set to 0.4 kJ/mol and was added every 2 ps; the Gaussian width was set to 0.35 nm; the bias factor was set to 10. Other simulation parameters and conditions are the same as those in the standard MD simulations. The well-tempered metadynamics simulations were run for 100 ns, followed by FEL constructions using the weighted histogram analysis method implemented in Plumed [83].

## 5. Conclusions

By performing multiple, long MD simulations on the proteinase K-solvent system with the protein and solvent coupled to either 300 or 180 K, we demonstrate that the temperatures of the solvent, when compared to the temperatures of the protein itself, have an overwhelming effect on protein dynamics. The flexibility, mobility, global collective motions, and sampled conformational spaces of proteinase K are greatly enhanced (or suppressed) by elevating (or lowering) the solvent temperature, whereas the changes of the protein temperature exert relatively weak effect on the above properties. The constructed FELs reveal that proteinase K has more conformational substates, larger conformational entropy, and lower thermostabilility at the high than at the low solvent temperatures, but these thermodynamic properties are independent of the protein temperatures. Elevating the solvent temperature increases the mobility/kinetic energy of the water molecules. By comparing the dynamic HB numbers in the MD trajectories at different combined temperatures, we demonstrate that it is the competitive interactions between water molecules and surface-exposed protein atoms and between atoms within the protein that transmit the solvent kinetic energy over the entire protein structure, ultimately leading to enhanced conformational flexibility and collective motions of the protein at the high solvent temperatures. Finally, we propose a refined FEL model to explain the role of solvent mobility in facilitating the cascade amplification of microscopic motions of atoms and atomic groups into the protein global collective motions.

## Figures and Tables

**Table 1:** Average structural properties (standard deviations are in parentheses) of proteinase K calculated from the joined MD trajectories at the four combined temperatures.

Temp ^a^ (K)	RMSD ^b^ (Å)	SASA ^c^ (Å^2^)	NNC ^d^	Rg ^e^ (Å)	SSE ^f^		
					α-Helix	β-Sheet	Turn
P180/S180	0.62 (0.03)	10,574 (96)	137,766 (474)	16.6 (0.3)	69.9 (1.6)	62.0 (2.4)	42.7 (3.5)
P300/S180	0.68 (0.04)	10,679 (136)	136,887 (620)	16.6 (0.3)	69.6 (1.6)	61.4 (2.7)	40.0 (3.6)
P180/S300	0.90 (0.08)	10,767 (162)	136,502 (756)	16.7 (0.5)	69.7 (1.7)	59.2 (3.7)	40.0 (4.7)
P300/S300	1.24 (0.18)	10,972 (192)	134,821 (947)	16.7 (0.5)	69.6 (1.9)	57.7 (5.1)	32.4 (5.0)

^a^ Temperatures of the protein (P) and the solvent (S); ^b^ Root mean square deviation of the backbone atoms with respective to the starting structure; ^c^ Total solvent accessible surface area; ^d^ Number of native contacts. A native contact is considered to exist if the distance between two atoms is less than 6 Å; ^e^ Radius of gyration; ^f^ Number of residues in the corresponding secondary structural elements.

**Table 2:** Properties of HBs within proteinase K calculated from the joined MD trajectories at the four combined temperatures.

Temp ^a^ (K)	Number ^b^					Persistency ^c^ (%)			
	Stat ^d^	Dyna ^e^	M–M ^f^	M–S ^g^	S–S ^h^	Dyna ^e^	M–M ^f^	M–S ^g^	S–S ^h^
P180/S180	239.2 (5.3)	540	217	215	108	44.3	66.7	25.7	36.1
P300/S180	232.9 (6.1)	712	262	294	156	30.7	49.3	17.7	23.9
P180/S300	229.0 (6.8)	1121	297	515	309	18.8	42.0	10.1	11.2
P300/S300	215.6 (7.8)	1987	469	979	539	9.4	23.5	4.5	5.8

^a^ Temperatures of the protein (P) and the solvent (S); ^b^ Number of the corresponding HBs; ^c^ Average HB persistency of the corresponding HBs; ^d^ Average static HBs in each frame. Standard deviations are in parentheses; ^e^ Dynamic HBs in a trajectory; ^f^ Main chain-main chain HBs in a trajectory; ^g^ Main chain-side chain HBs in a trajectory; ^h^ Side chain-side chain HBs in a trajectory.

**Table 3:** Properties of HBs formed between proteinase K and the solvent calculated from the joined MD trajectories at the four combined temperatures.

Temp ^a^ (K)	Number ^b^				Persistency ^c^ (%)		
	Stat ^d^	Dyna ^e^	M–Solv ^f^	S–Solv ^g^	Dyna ^e^	M–Solv ^f^	S–Solv ^g^
P180/S180	391.3 (11.7)	8475	627	7848	4.62	8.34	4.32
P300/S180	377.2 (11.7)	17,960	1435	16,525	2.10	3.34	1.99
P180/S300	363.8 (14.3)	743,864	74,319	669,545	0.05	0.07	0.05
P300/S300	330.4 (14.8)	1,034,810	120,772	914,038	0.03	0.03	0.03

^a^ Temperatures of the protein (P) and the solvent (S); ^b^ Number of the corresponding HBs; ^c^ Average HB persistency of the corresponding HBs; ^d^ Average static HBs in each frame. Standard deviations are in parentheses; ^e^ Dynamic HBs in a trajectory; ^f^ Main chain-solvent HBs in a trajectory; ^g^ Side chain-solvent HBs in a trajectory.

## Supplementary Materials

Supplementary materials can be found at http://www.mdpi.com/1422-0067/17/2/254/s1.
