# Supplementary material for: Effect of the Solvent Temperatures on Dynamics of Serine Protease Proteinase K
Source: Int J Mol Sci. 2016 Feb 19;17(2):254. doi: 10.3390/ijms17020254 (PMC4783983; doi:10.3390/ijms17020254)
Supplement: Supplementary file 1 [file ijms-17-00254-s001.pdf]

Supplementary Materials: Effect of the Solvent Temperatures on Dynamics of Serine Protease Proteinase K

Peng Sang, Qiong Yang, Xing Du, Nan Yang, Li-Quan Yang, Xing-Lai Ji, Yun-Xin Fu, Zhao-Hui Meng and Shu-Qun Liu

Table S1. Cosine contents of the first three eigenvectors (eig.1–eig.3) calculated from the six independent equilibrated MD trajectories (1–15 ns; traj. 1–6) and the single joined (84 ns; traj. joined) trajectories. The lower cosine contents of eig.1–eig.3 of the joined trajectories than the corresponding values of traj. 1–6 suggest that a higher degree of sampling convergence can be achieved when the joined trajectories are considered.

traj.	P180/S180			P300/S180			P180/S300			P300/S300		
	eig.1	eig.2	eig.3	eig.1	eig.2	eig.3	eig.1	eig.2	eig.3	eig.1	eig.2	eig.3
1	0.9122	0.8407	0.8192	0.8822	0.5805	0.3693	0.6077	0.6365	0.3091	0.9216	0.2920	0.2704
2	0.9326	0.8722	0.6860	0.6798	0.6043	0.5874	0.8697	0.4236	0.2013	0.4960	0.4009	0.5886
3	0.8377	0.7256	0.7028	0.8040	0.6687	0.6430	0.8425	0.3582	0.1309	0.7968	0.7758	0.2893
4	0.8948	0.7842	0.5395	0.8790	0.4998	0.5010	0.5182	0.5969	0.5310	0.5045	0.3155	0.5084
5	0.8621	0.7708	0.7291	0.8839	0.7294	0.5360	0.6762	0.1280	0.1480	0.8934	0.4284	0.2860
6	0.8978	0.7666	0.7926	0.8262	0.8173	0.6450	0.6947	0.3168	0.1940	0.9226	0.3543	0.5137
joined	0.0060	0.2375	0.1564	0.2461	0.2038	0.1040	0.2325	0.0186	0.0063	0.1124	0.0119	0.0178

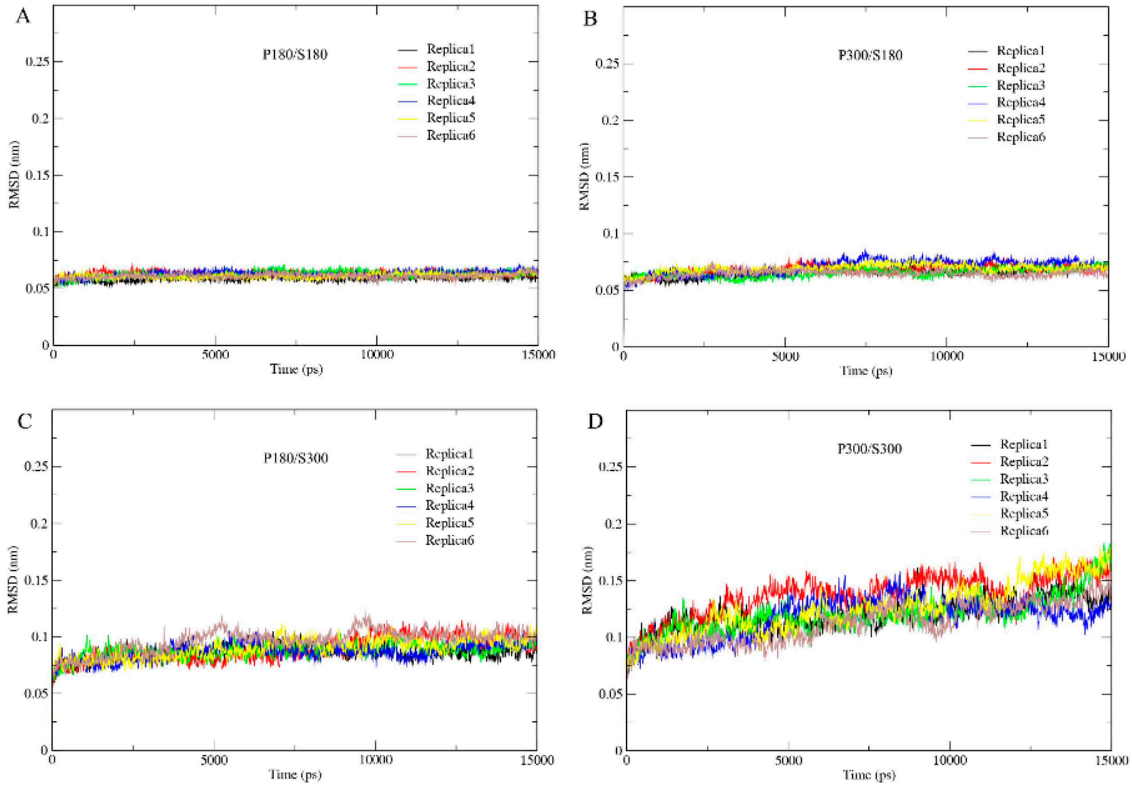

Figure S1. Time-dependent backbone RMSD values of proteinase K with respect to the starting structure during the 6 independent MD simulations (replicas 1–6). (**A**) P180/S180; (**B**) P300/S180; (**C**) P180/S300; (**D**) P300/S300.

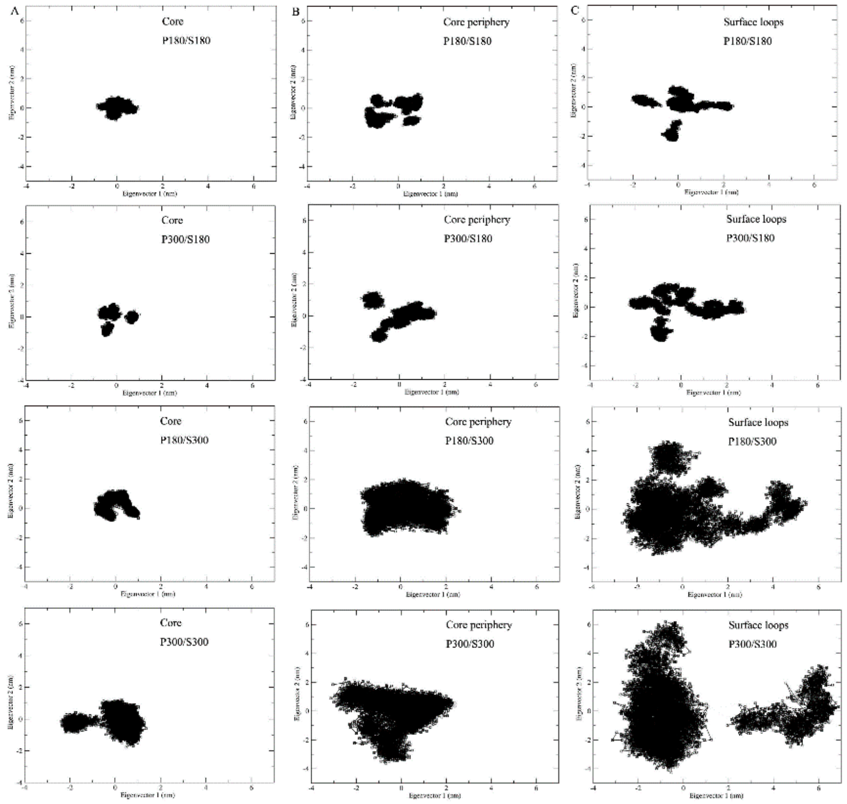

Figure S2. Projections of MD trajectories of different structural parts onto the two-dimensional conformational subspaces spanned by the first two eigenvectors. (**A**–**C**) are conformational space regions explored by the protein core, core periphery, and surface loops, respectively, at the four combined temperatures which are arranged from top to bottom in the order P180/S180, P300/S180, P180/S300, and P300/S300.

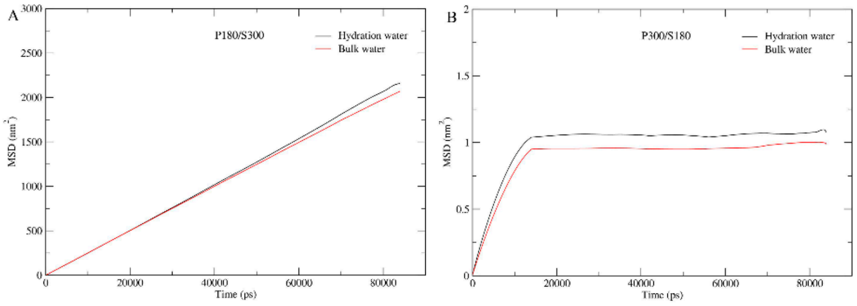

Figure S3. Time evolution of mean square displacements (MSDs) of the hydration water and the bulk water calculated from the joined MD trajectories of (**A**) P180/S300 and (**B**) P300/S180. In our calculation the hydration water is defined as the water molecules within 5 Å from the protein surface in the starting simulation system. Under the P180/S300 combination (**A**), the observed higher MSD of the hydration water than the bulk water at lag-times longer than ~30 ns could be due to (i) the gradual diffusion of the initially defined hydration water molecules away from the protein surface making them finally become a part of the bulk water; and (ii) smaller population size of the hydration water than the bulk water. The diffusion coefficient (10–5 cm^2/s) evaluated from 0–5 ns MSD curve is 3.779 ± 0.011 and 3.963 ± 0.049 for the hydration water and bulk water, respectively, indicating a lower diffusion capacity of the hydration water than bulk water when it is located close to the protein surface. At P300/S180 (**B**), both the hydration water and bulk water are in a structurally arrested, glass-like state, with the former demonstrating slightly higher MSD values than the latter due to the physical contacts between the protein (which are in the high temperature) and the hydrated water molecules.
